# A survey of the awareness, knowledge, policies and views of veterinary journal Editors-in-Chief on reporting guidelines for publication of research

**DOI:** 10.1186/1746-6148-10-10

**Published:** 2014-01-10

**Authors:** Douglas JC Grindlay, Rachel S Dean, Mary M Christopher, Marnie L Brennan

**Affiliations:** 1 Centre for Evidence-based Veterinary Medicine, School of Veterinary Medicine and Science, The University of Nottingham, Sutton Bonington Campus, Loughborough LE12 5RD, UK; 2 School of Veterinary Medicine, 4206 VM3A, University of California–Davis, One Shields Ave, Davis, CA 95616, USA

**Keywords:** Veterinary journals, Veterinary research, Reporting guidelines, Reporting quality, Editors, Editorial policies, Views, Barriers

## Abstract

**Background:**

Wider adoption of reporting guidelines by veterinary journals could improve the quality of published veterinary research. The aims of this study were to assess the knowledge and views of veterinary Editors-in-Chief on reporting guidelines, identify the policies of their journals, and determine their information needs. Editors-in-Chief of 185 journals on the contact list for the International Association of Veterinary Editors (IAVE) were surveyed in April 2012 using an online questionnaire which contained both closed and open questions.

**Results:**

The response rate was 36.8% (68/185). Thirty-six of 68 editors (52.9%) stated they knew what a reporting guideline was before receiving the questionnaire. Editors said they had found out about reporting guidelines primarily through articles in other journals, via the Internet and through their own journal. Twenty of 57 respondents (35.1%) said their journal referred to reporting guidelines in its instructions to authors. CONSORT, REFLECT, and ARRIVE were the most frequently cited. Forty-four of 68 respondents (68.2%) believed that reporting guidelines should be adopted by all refereed veterinary journals. Qualitative analysis of the open questions revealed that lack of knowledge, fear, resistance to change, and difficulty in implementation were perceived as barriers to the adoption of reporting guidelines by journals. Editors suggested that reporting guidelines be promoted through communication and education of the veterinary community, with roles for the IAVE and universities. Many respondents believed a consensus policy on guideline implementation was needed for veterinary journals.

**Conclusions:**

Further communication and education about reporting guidelines for editors, authors and reviewers has the potential to increase their adoption by veterinary journals in the future.

## Background

Reporting guidelines, standards or statements (referred to here as reporting guidelines) can be defined as “statements that provide advice on how to report research methods and findings” [1]. The aim of reporting guidelines is to improve the transparency, accuracy and completeness of reporting for different types of research studies, ultimately improving the reliability and value of published research [1,2]. Reporting guidelines have been implemented by many medical journals and there have been calls for wider adoption of these tools [3,4]. Several studies have found that the quality of reported research was improved in journals that had adopted reporting guidelines [5-8].

Around 200 reporting guidelines are listed on the website of the EQUATOR Network [9], which promotes their development and dissemination. Popham and colleagues [10] identified five “core” reporting guidelines relating to major research designs: CONSORT for randomised controlled trials [11], TREND for non-randomised controlled trials [12], STROBE for observational studies in epidemiology [13], PRISMA for systematic reviews and meta-analyses [14] and STARD for studies of diagnostic accuracy [15]. Two additional reporting guidelines are specifically relevant for veterinary medicine: REFLECT for randomised controlled trials for livestock and food safety [16] and ARRIVE for research using laboratory animals [17]. The International Association of Veterinary Editors (IAVE) has also published a consensus reporting guideline on animal ethics and welfare that addresses the use of client-owned animals [18].

Several authors have demonstrated deficiencies in the reporting of study design features and other methodological information necessary to judge the quality of the evidence in veterinary research, especially in clinical trials [19-23]. For more than a decade, commentaries have called for the adoption of reporting guidelines by veterinary journals to improve the quality of published research and enable more effective critical appraisal [2,24-33]. More [2] recommended that veterinary journals "require author compliance” with relevant reporting guidelines. However, the general awareness of veterinary journal editors about reporting guidelines and the extent to which veterinary journals have implemented reporting guidelines is unknown.

EQUATOR is currently investigating the factors that prevent or facilitate the use of reporting guidelines by medical journals [3]. Corresponding information is lacking on potential barriers and facilitating factors in the implementation of reporting guidelines by veterinary journals. The aims of this study were to assess the knowledge and views of Editors-in-Chief of veterinary journals about reporting guidelines, and to identify current and planned journal policies on the implementation of reporting guidelines. A further aim was to assess the information needs of editors in relation to reporting guidelines, and how these could best be met.

## Methods

This research received ethical approval from the School of Veterinary Medicine and Science Ethics Committee at The University of Nottingham. As stated in the introductory letter of the online questionnaire (Additional file 1), consent to participate in the research was implied by completion of the questionnaire. No incentives were offered for participation. In reporting this study, reference was made to the checklist of items in the STROBE statement [13].

### Sample selection

The reference population for this cross-sectional study was Editors-in-Chief of veterinary journals and of animal science journals that publish papers of relevance to veterinary medicine and science. The sampling frame was Editors-in-Chief of journals on the contact list for the IAVE for whom a valid e-mail address was available. This contact list is maintained by the Organising Chair of the IAVE (MC), through a combination of direct communication with veterinary editors and information obtained from bibliographic databases and journal websites.

The IAVE e-mail contact list used for the survey invitation was updated as of 1^st^ April 2012, and included Editors-in-Chief from 197 journals. For journals with more than one Editor-in-Chief, the editors were asked in both the invitation e-mail and the introductory letter to return a single, joint response for their journal.

### Questionnaire development

The questionnaire was made anonymous to encourage full and accurate responses; however respondents had the option to provide identifying information at the end of the questionnaire. The introductory letter explained the purpose of the study and listed the investigators involved.

The online questionnaire was developed and hosted using Cvent Web Survey software (Cvent, Inc., McLean, VA, USA; http://www.cvent.com). The text of the questionnaire is shown in Additional file 1. The questionnaire included closed, semi-closed and open questions that were organized in the following five sections:

Section 1 - The respondent’s present knowledge of reporting guidelines;

Section 2 - Current and planned implementation of reporting guidelines by the respondent’s journal;

Section 3 - The respondent’s views on the potential need for reporting guidelines and their implementation by veterinary journals;

Section 4 - The respondent’s information needs concerning reporting guidelines;

Section 5 - Optional identifying information, including the country the journal was published in and the name of the journal.

The first question in the questionnaire (“Before receiving this questionnaire, did you know what a reporting guideline/standard was?”) was compulsory. Respondents answering “Yes” to this question were asked how and where they had learned about reporting guidelines and which reporting guidelines they were aware of, from a list of options. Respondents answering “No” were directed to Section 2 and the next compulsory question for all respondents, on whether their journal’s instructions to authors mentioned reporting guidelines. Different questions were posed to respondents whose journal already referred to reporting guidelines than to those whose journal did not. At the start of Section 2, a brief definition of reporting guidelines was given to inform any respondents previously unaware of what they were. Respondents were instructed to consult their journal’s editorial team if they lacked the information to answer the questions on their journal’s current and planned implementation of reporting guidelines.

The questionnaire was pre-tested by seven researchers from the Centre for Evidence-based Veterinary Medicine and then piloted online by three Assistant Editors and a former Editor-in-Chief of veterinary journals, and five veterinary researchers from the University of Nottingham. It was ensured that the logic of the online questionnaire functioned correctly, and any ambiguous or misunderstood questions were re-worded.

### Questionnaire distribution

Editors-in-Chief on the IAVE contact list were e-mailed an invitation to take part in the survey by MC on behalf of the IAVE on 18 April 2012. The invitation included a brief description of the study and a link to the online questionnaire. If an e-mail failure message was received after sending out the initial invitation, the e-mail was resent to the same address two days later. If the e-mail failed a second time, an attempt was made to find an alternative e-mail address by searching the journal’s website, and the invitation was resent if another address was found. Reminders were sent to all recipients after two weeks and on the day before the questionnaire closed. The closing date was 16 May 2012, four weeks after the initial invitation was sent.

### Analysis of data

Identifying details from the optional Section 5 of the questionnaire were removed from each response before analysis, to anonymise the data.

Depending on the web browser used and the settings for cookies, the Cvent software used the internet protocol (IP) address to identify where a respondent had partially completed the questionnaire and then returned later. In such cases the answers initially entered were retained, and a single respondent identification number was generated by the software. To check for potential failures in this automatic process, the IP address of each response was also checked manually for duplicates.

Data were exported from the Cvent website to an Excel spread sheet (Microsoft Corp, Redmond, WA, USA) and descriptive analysis was performed. All categorical variables were reported as numbers and percentages. Responses for any open or semi-closed questions that required a simple, factual answer (e.g. sources of information about reporting guidelines) were coded by one author (DG). For open questions that produced complex, free-text responses where quantitative analysis was inappropriate, DG and one other author (RD or MB) undertook independent qualitative analyses to identify themes in the data [34] and code the responses. Agreement on the themes and coding was then reached by discussion where necessary. Hence, these results are reported in a descriptive, and not a numerical, fashion.

## Results

### Response rate

From the 197 e-mail addresses on the IAVE list, 16 produced an e-mail failure message after both the first and second attempts. Alternative e-mail addresses were found for eight of these journals and the invitation e-mail resent, but e-mail failure messages were still received for four journals. Hence, it was presumed that the Editors-in-Chief of 185 journals received the e-mail invitation (Appendix 1).

A total of 76 responses were received. Eight responses were identified from the IP address as partial responses where respondents had subsequently answered the questionnaire in full without this being automatically recognised by the Cvent software, so these partial responses were removed from the sample. Therefore 68 usable responses were received from the Editors-in-Chief of the 185 journals contacted, giving a response rate of 36.8%.

### Characteristics of respondents

Optional information on the country of journal publication was provided by 37 of 68 respondents (54.4%). Seventeen journals were from Europe, eight from North America, five from Asia, six from Africa and one from Australia. The title of their journal was provided by 34 of 68 respondents (50.0%).

### Previous knowledge of reporting guidelines

Just under 53% (36/68) of total respondents knew what a reporting guideline was before receiving the questionnaire, while 47.1% (32/68) had no previous knowledge. Thirty-two of the 36 respondents who knew what a reporting guideline was answered some or all of the supplementary questions that were presented to them in Section 1 of the questionnaire. These editors had mainly learned about reporting guidelines from articles in other journals, by the Internet and through their own journal, with several naming more than one source (Table 1). Of this group, 31.3% (10/32) said they were aware of the EQUATOR Network and its resources on reporting guidelines, compared to 62.5% (20/32) who were not aware. Seventy-five per cent (24/32) were aware of separate reporting guidelines for different types of studies, and 18.8% (6/32) were not. Twenty-three respondents specified the guideline(s) with which they were familiar from a supplied list of twelve (Table 2), with CONSORT, ARRIVE and REFLECT being the most cited.

**Table 1:** Information sources where Editors-in-Chief of veterinary journals (n = 32) learned about reporting guidelines

**Information source**	Number of respondents*	Percentage of respondents*
Other journals	10	31.3
Internet	7	21.9
Own journal, including editorial discussions	6	18.8
Medical literature	3	9.4
Professional colleagues	3	9.4
International Association of Veterinary Editors (IAVE)	2	6.3
Postgraduate study	2	6.3
Other sources (one respondent each)†	11	34.4

*Numbers and percentages add up to more than 32 and 100% respectively because some respondents named more than one source.

†Other sources were: books, Committee on Publication Ethics (COPE), EQUATOR Network, European Food Safety Authority (EFSA), International Committee of Medical Journal Editors (ICMJE), Journal club, lectures, MEDLINE, National Institutes of Health (NIH), publishers, and reporting guideline authors.

**Table 2:** Awareness of specific reporting guidelines among Editors-in-Chief of veterinary journals with previous knowledge of reporting guidelines (n = 23)

**Reporting guideline**	Number of respondents*	Percentage of respondents*
CONSORT (randomised controlled trials/RCTs)	20	87.0
ARRIVE (research using laboratory animals)	16	69.6
REFLECT (RCTs for livestock and food safety)	12	52.2
STARD (diagnostic accuracy studies)	9	39.1
TREND (non-randomised controlled trials)	9	39.1
PRISMA (systematic reviews and meta-analyses)	8	34.8
COGS (clinical guidelines)	7	30.4
Gold Standard Publication Checklist (animal research)	6	26.1
STROBE (observational studies)	6	26.1
COREQ (qualitative research)	4	17.4
MOOSE (meta-analyses of observational studies in epidemiology)	3	13.0
STREGA (genetic association studies)	2	8.7

*Numbers and percentages add up to more than 23 and 100% respectively because some respondents were aware of more than one reporting guideline.

### Current journal policies and future plans

Fifty-seven respondents (83.8% of total) answered the second compulsory question on whether their journal referred to any reporting guidelines in its instructions to authors. Of these, 35.1% (20/57) said that their journal did, 59.6% (34/57) said that their journal did not, and 5.3% (3/57) said they did not know.

Sixteen of the 20 respondents whose journals referred to reporting guidelines indicated the guidelines used (Table 3). In addition to those reporting guidelines listed in the questionnaire, one respondent specified the ORION statement for studies of outbreaks and nosocomial infections [35], and two respondents cited the International Committee of Medical Journal Editors (ICMJE) *Uniform Requirements for manuscripts submitted to biomedical journals *[36]. Five respondents cited only the author guidelines for their own journal rather than consensus reporting guidelines for different study types. Nine (45%) of the 20 respondents whose journal referred to reporting guidelines said they had plans to implement additional reporting guidelines in the future.

**Table 3:** Reporting guidelines mentioned in the instructions to authors of veterinary journals (n = 16)

**Reporting guideline**	Number of journals*	Percentage of respondents*
ARRIVE (research using laboratory animals)	4	25.0
CONSORT (randomised controlled trials/RCTs)	4	25.0
REFLECT (RCTs for livestock and food safety)	4	25.0
PRISMA (systematic reviews and meta-analyses)	3	18.8
STARD (diagnostic accuracy studies)	3	18.8
STROBE (observational studies)	3	18.8
ICMJE (Uniform Requirements for Manuscripts Submitted to Biomedical Journals)	2	12.5
ORION (outbreak reports and intervention studies of nosocomial infection)	1	6.3

*Numbers and percentages add up to more than 16 and 100% respectively because some journals referred to more than one reporting guideline.

Fifteen respondents whose journals referred to reporting guidelines in their instructions to authors indicated what they did with submitted studies that did not follow the relevant reporting guideline, but otherwise satisfied their editorial criteria. Three editors said they would be rejected, 11 said they would be returned to the authors to modify in line with the reporting guideline, and one editor said they did nothing, but relied on the reviewers’ comments.

For respondents whose journal did not refer to reporting guidelines, the reasons given included the opinion that existing instructions to authors and review processes were sufficient, and that reporting guidelines were not appropriate for the types of article submitted to their journal. Lack of personal knowledge or previous consideration of reporting guidelines by the editors themselves, the setting of policy by the publisher, and the belief that reporting was a responsibility for authors were other themes in the data. It was also suggested that existing reporting guidelines were difficult to implement and would place an undue burden on reviewers.

Respondents whose journal did not refer to reporting guidelines in their instructions to authors were asked if their journal had plans to implement reporting guidelines in the future. Of these respondents, 29.4% (10/34) responded “Yes”, 17.6% (6/34) responded “No”, 35.3% (12/34) responded “Don’t know”, and 17.6% (6/34) did not respond.

### Views on the need to implement reporting guidelines

Forty-four respondents (65.7% of total) answered the question on whether they believed that reporting guidelines should be adopted by all refereed veterinary journals. Of these, 68.2% (30/44) believed they should, 11.4% (5/44) believed they should not, and 20.5% (9/44) said they did not know.

In response to the open questions, the main reasons given by those believing they should be adopted by all refereed veterinary journals were that guidelines would improve the quality of reporting of research, would ensure consistency of standards across studies and across journals, and provide a guide to reviewers and authors. One respondent commented: “If all journals started to emphasise reporting, ultimately authors would start to consider these aspects in their study design at the planning stage”.

The themes arising from the responses of those who thought reporting guidelines should not be adopted by all refereed veterinary journals included the belief that the current system was satisfactory, that there should be “a freedom for editors and authors to have other ways of ensuring high quality work”, that reporting guidelines did not suit all subject areas or study types, and that authors might be hesitant to submit to journals that applied the guidelines strictly.

### Factors preventing the adoption of reporting guidelines

The most common theme arising in responses to the open question about factors preventing the adoption of reporting guidelines was a lack of knowledge among authors, reviewers and editors. The influence of “tradition”, fear or resistance to change within the veterinary community was also widely mentioned, as well as concern about cultural differences between journals and between countries, the increased workload that could be caused, and economic factors. Another theme was the belief that authors would prefer journals without reporting guidelines, leading to a fear of losing submissions if a journal were to adopt them. One editor referred to “the current need to accept/publish minor quality papers even in higher ranking veterinary journals”, and another referred to the “publish or perish syndrome” as potential factors preventing the adoption of reporting guidelines.

### Factors or actions that would promote more widespread adoption of reporting guidelines

A common theme on factors to promote more widespread adoption of reporting guidelines was communication and increased awareness amongst the veterinary community, with roles proposed for the IAVE, EQUATOR Network and Centre for Evidence-based Veterinary Medicine. Suggested ways to increase awareness included promotion on the Internet, information and links on journal websites, editorials, conferences and targeting authors from developing countries. The importance of education and continuing professional development for all involved in the authoring and publishing process was highlighted, including a specific role for universities in educating veterinary students and young researchers about reporting guidelines. It was suggested that there should be a consensus policy among veterinary journals on guideline implementation, with the IAVE involved. There were conflicting opinions on who should “enforce” guidelines, whether editors or authors, through “pressure” on journals.

Several respondents indicated that this survey had in itself made them aware of reporting guidelines. One respondent commented: “It made me think about several aspects of our journals and how can we improve our researchers’ capability to report accurately their findings”.

### Information needs

Of the 42 editors who answered the question on whether more information about reporting guidelines would be useful, 37 (88.1%) said “Yes” and five (11.9%) said “No”. In the open responses, the most suggested ways to disseminate information on reporting guidelines to veterinary editors were by e-mail, meetings, and websites. Editorials, articles and educational activities were also mentioned. Again several respondents indicated that the IAVE should play a part.

## Discussion

Major findings of this study were that nearly half of the responding Editors-in-Chief had not previously heard about reporting guidelines, and that only around a third of their journals referred to reporting guidelines in their instructions to authors. This lack of awareness is likely to be a barrier to their widespread implementation by veterinary journals.

Among the Editors-in-Chief who were aware of reporting guidelines, CONSORT, REFLECT, and ARRIVE were the most widely-known, and these were also the most frequently mentioned guidelines in instructions to authors according to the survey. This may reflect the types of studies published in many veterinary journals (randomised controlled trials and experimental animal studies) or the ways in which information about these guidelines has been disseminated. CONSORT is one of the most widely cited reporting guidelines in the medical literature and has undergone evaluation and revision over time [37]. There have been several commentaries in veterinary journals focusing on CONSORT [24,25,30,32], REFLECT [30,32,38] and ARRIVE [39]. Such promotion is likely to have contributed to awareness and implementation of these guidelines by veterinary editors.

Although their journal’s instructions to authors were considered as a type of reporting guideline by five editors in this survey, author guidelines do not usually provide the detailed information necessary for complete and accurate reporting of individual study types, and do not reflect a consensus among experts. Two editors cited the ICMJE *Uniform Requirements *[36] as their journal’s reporting guidelines, but this document mainly addresses editing and ethical issues rather than reporting. If these seven respondents are excluded from our analysis, the proportion of veterinary journals that referred to reporting guidelines decreases from 35.1% to 22.8%.

Implementation of guidelines by journals can occur in several ways—inclusion in instructions to authors, inclusion in instructions to reviewers, and the use of reporting guideline checklists for authors and referees [28,40]. There is also the possibility of editors rejecting papers that do not meet the relevant guideline [2,28], although Vandenbroucke [40] suggested it may not be practical for editors to do the checking. It is therefore noteworthy that in this survey almost all the Editors-in-Chief whose journal referred to reporting guidelines in author instructions said that non-complying articles would be rejected or returned to authors for amendment.

A recent systematic review found that medical journal endorsement of CONSORT was insufficient to ensure the completeness of reporting [37], and two studies found that the quality of articles on studies of diagnostic accuracy did not notably improve when STARD was included in author guidelines, largely because of lack of adherence to those guidelines [41,42]. In another study, authors found it difficult to adhere to high methodological standards after the research had already been done [43]. This highlights the importance of undergraduate and postgraduate education on reporting guidelines, as suggested in our survey and elsewhere [26,28]. Reporting guidelines also appear to be underutilised by peer reviewers for medical journals [44], and reviewers and editors sometimes overlook lack of adherence to reporting guidelines to publish for other reasons [41]. Thus, it will be important to monitor the methods and impact of implementation if more veterinary journals adopt reporting guidelines in the future.

It is notable that some respondents believed that reporting guidelines were not necessary, or that their own guidelines and review process were enough to ensure the quality of published papers. Yet analyses of published veterinary research suggest there is room for improvement [19-23] and the quality of reported research has been improved in medical journals where reporting guideline checklists were utilised [5-8]. There were also editors in this survey who expressed the fear that reporting guidelines would limit the freedom of journals and authors, and that enforcement would mean a loss of authors and manuscripts to other journals. Such concerns and resistance to reporting guidelines have been echoed by editors of medical journals [40]. Gradual implementation of reporting guidelines could give authors the chance to improve their study design in anticipation of reporting requirements. Another solution, proposed by respondents in this survey, would be to achieve a consensus on the implementation of reporting guidelines among veterinary journals, as has also been suggested for medical journals [40].

Our survey results indicate that two-thirds of Editors-in-Chief believed that reporting guidelines should be adopted by all peer-reviewed journals, and many expressed an interest in receiving more information about reporting guidelines. Thus, more effective dissemination of information to editors and authors and a better understanding of the relevance of reporting guidelines to veterinary research have strong potential to increase their adoption by veterinary journals in the future. A relatively high proportion of respondents learned about reporting guidelines through other journals, so editorials and the re-publication of reporting guidelines appear to be effective ways of disseminating information about their use and applicability in different disciplines. Many respondents mentioned the Internet as a source of information—there is scope to increase awareness of the resources for editors on the EQUATOR Network [9] and IAVE [45] websites. Similar findings about the need for dissemination of information to editors, including education and provision of electronic resources, were found in a recent survey of medical journal editors on implementation of the CONSORT guidelines [46]. A more proactive approach to dissemination seems warranted, including direct contact with veterinary editors. This survey has in itself been part of the process, according to some of the participants.

### Limitations of the study

The response rate of 36.8% for this survey was comparable to the response rates of 27.6% and 39% obtained by Shamseer et al. [46] and Hopewell et al. [47] respectively in their surveys of medical journal editors about CONSORT. One possible reason for non-responses in this survey could have been language, as many journals in the IAVE list (Appendix 1) are based in non-English speaking countries and are not published in English. Reporting guidelines are also heavily focused on epidemiologic study design and clinical medicine, which are not well-developed disciplines in veterinary medicine in all countries. Another potential reason for non-responses is that some veterinary journals are not peer-reviewed or do not publish original research, so reporting guidelines would not be so relevant for their editors.

A possible limitation to this study is that survey recipients with an awareness of, or interest in, reporting guidelines might be more likely to respond. The results might therefore have been questionable if the survey had found a high degree of awareness of reporting guidelines among the respondents. However, it is clear from the actual data that there were considerable numbers of Editors-in-Chief who were previously unaware of reporting guidelines, with potentially more among the non-respondents.

Another potential issue is that it was possible for respondents to go back and change their answer to Question 1, “Before receiving this questionnaire, did you know what a reporting guideline/standard was”, after they saw the explanation of reporting guidelines later in the questionnaire. Some respondents may not have answered truthfully if they thought this was knowledge they should possess. However, the answers to Question 1 could also have been refined in cases where respondents were familiar with the concept of reporting guidelines, but did not initially recognise the term in Question 1.

As the questionnaire could be answered anonymously, it was not possible to obtain information about all respondents and to determine whether they were representative of the population of journals in the IAVE database. Anonymity was felt to be important to ensure an adequate response rate, because the survey was partly about the editors’ personal knowledge and views on a potentially sensitive topic. Indeed, since only around half of the responding editors chose to identify themselves, this suggests that the response rate would have been lower had disclosure been required. Importantly, those Editors-in-Chief who did provide identifying details represented a wide range of geographical locations.

This survey is subject to reporting bias, as respondents were asked to report what they do, which may not be what they actually do in practice. Only an examination of the instructions to authors for all the journals in the sampling frame would provide a completely accurate assessment of current practice.

## Conclusions

The results of this survey provide valuable information on the awareness, knowledge, policies, and views of veterinary journal Editors-in-Chief about reporting guidelines, and perceived barriers to their adoption by veterinary journals. Editors have an important leadership role in the implementation of reporting guidelines and improving the quality of research reporting, yet many appear to have little or no knowledge of reporting guidelines. More effective communication and education about reporting guidelines for editors, reviewers and authors has strong potential to increase their adoption by veterinary journals in the future.

## Appendix

### Appendix 1 – Journals on the International Association of Veterinary Editors (IAVE) e-mail list whose Editors-in-Chief were presumed to have received the survey invitation (n = 185)

Acta Scientiae Veterinariae

Acta Veterinaria (Beograd)

Acta Veterinaria Brno

Acta Veterinaria Hungarica

Acta Veterinaria Scandinavica

Advances in Small Animal Medicine and Surgery

Alexandria Journal of Veterinary Sciences

Anatomia, Histologia, Embryologia

Animal

Animal Behaviour

Animal Biotechnology

Animal Genetics

Animal Health Research Reviews

Animal Reproduction Science

Animal Welfare

Annales de Médecine Vétérinaire

Anthrozöos

Archives of Veterinary Science

Archivos de Medicina Veterinaria

Arquivo Brasileiro de Medicina Veterinária e Zootecnia

Australian Cattle Veterinarians

Australian Veterinary Journal

Avian Diseases

Avian Pathology

Berliner und Münchener Tierärzliche Wochenschrift

BMC Veterinary Research

Brazilian Journal of Veterinary Research and Animal Science

Bulgarian Journal of Veterinary Medicine

Bulletin of Animal Health and Production in Africa

Canadian Journal of Veterinary Research

Canadian Veterinary Journal

Ciência e Agrotecnologia

Clinician's Brief

Comparative Clinical Pathology

Comparative Exercise Physiology

Comparative Immunology, Microbiology, and Infectious Diseases

Comparative Medicine

Danish Veterinary Journal/Dansk Veterinærtidsskrift

Diseases of Aquatic Organisms

Domestic Animal Endocrinology

Egyptian Journal of Comparative Pathology and Clinical Pathology

Egyptian Journal of Sheep and Goat Sciences

Equine Veterinary Education

Equine Veterinary Journal

Estonian Veterinary Review

European Journal of Companion Animal Practice

Finnish Veterinary Journal/Suomen Eläinlääkärilehti

Flemish Veterinary Journal/Vlaams Diergeneeskundig Tijdschrift

Folia Veterinaria

Hungarian Veterinary Journal/Magyar Âllatorvosok Lapja

ILAR Journal

Indian Veterinary Journal

International Journal of Applied Research in Veterinary Medicine

International Journal of Veterinary Research

Internet Journal of Veterinary Medicine

Ippologia

Iranian Journal of Veterinary Research

Iraqi Journal of Veterinary Sciences

Irish Veterinary Journal

Israel Journal of Veterinary Medicine

Japanese Journal of Veterinary Research

Journal of Advanced Research

Journal of the American Animal Hospital Association

Journal of the American Veterinary Medical Association / American Journal of Veterinary Research

Journal of Animal Breeding and Genetics

Journal of Animal Physiology and Animal Nutrition

Journal of Applied Animal Welfare Science

Journal of Aquatic Animal Health

Journal of Avian Medicine and Surgery

Journal of Camel Practice and Research

Journal of Comparative Pathology

Journal of the Egyptian Veterinary Medical Society of Parasitology

Journal of Equine Veterinary Science

Journal of Exotic Pet Medicine

Journal of Feline Medicine and Surgery

Journal of Fish Diseases

Journal of the Hellenic Veterinary Medical Society/Deltion tes Ellenikes Kteniatrikes Etaireias

Journal of Herpetological Medicine and Surgery

Journal of Medical Primatology

Journal of Small Animal Practice

Journal of the South African Veterinary Association

Journal of Swine Health and Production

Journal of Veterinary and Animal Sciences

Journal of Veterinary Behavior: Clinical Applications and Research

Journal of Veterinary Cardiology

Journal of Veterinary Dentistry

Journal of Veterinary Diagnostic Investigation

Journal of Veterinary Emergency and Critical Care

Journal of Veterinary Internal Medicine

Journal of Veterinary Medical Education

Journal of Veterinary Medical Science

Journal of Veterinary Pharmacology and Therapeutics

Journal of Veterinary Research

Journal of Veterinary Science

Journal of Veterinary Science and Technology

Journal of Wildlife Diseases

Journal of Wildlife Rehabilitation

Journal of Zoo and Wildlife Medicine

Kafkas Üniversitesi Veteriner Fakültesi Dergisi

Kafr-El-Sheikh Veterinary Medical Journal

Kenya Veterinarian

Kleintiermedizin

Kleintierpraxis

Laboratory Animals

Magazyn Weterynaryjny

Medical and Veterinary Entomology

Medycyna Weterynaryjna

New Zealand Veterinary Journal

Nigerian Veterinary Journal

Norwegian Veterinary Journal/Norsk Veterinærtidsskrift

Onderstepoort Journal of Veterinary Research

Online Journal of Veterinary Research

Pakistan Veterinary Journal

Pesquisa Veterinária Brasileira

Pferdeheilkunde

Philippine Journal of Veterinary and Animal Sciences

Pig Journal

Point Vétérinaire

Polish Journal of Veterinary Sciences

Praktische Tierarzt

Pratique Medicale et Chirurgicale de l'Animal de Compagnie

Preventive Veterinary Medicine

Production Animales (INRA)

Reproduction in Domestic Animals

Research in Veterinary Science

Revista de Ciências Agroveterinárias (Journal of Agronomy and Veterinary Sciences)

Revista do Centro de Ciências Rurais

Revista Mexicana de Ciencias Pecuarias

Revista Veterinária Notícias

Revue de Médecine Vétérinaire

Revue Scientifique et Technique (International Office of Epizootics)

Sahel Journal of Veterinary Sciences

Scandinavian Journal of Laboratory Animal Science

Schweizer Archiv für Tierheilkunde/SAT

Sheep and Goat Research Journal

Slovenian Veterinary Research

Small Ruminant Research

Suez Canal Veterinary Medical Journal

Swedish Veterinary Journal/Svensk Veterinärtidning

Tanzania Veterinary Journal

Thai Journal of Veterinary Medicine

Theriogenology

Tieraerztliche Praxis (Grosstiere)

Tieraerztliche Praxis (Kleintiere)

Tijdschrift voor Diergeneeskunde

Today's Veterinarian

Topics in Companion Animal Medicine

Transboundary and Emerging Diseases

Tropical Animal Health and Production

Tropical Veterinarian

Turkish Journal of Veterinary and Animal Sciences

Vet On-Line

Veterinaria México

Veterinarija ir Zootechnika

Veterinarski Arhiv

Veterinárství

Veterinary Anaesthesia and Analgesia

Veterinary and Comparative Oncology

Veterinary Bulletin

Veterinary Clinical Pathology

Veterinary and Comparative Orthopaedics and Traumatology

Veterinary Dermatology

Veterinary Economics

Veterinary Immunology and Immunopathology

Veterinary Journal

Veterinary Medicine: Research and Reports

Veterinary Medicine

Veterinary Microbiology

Veterinary Ophthalmology

Veterinary Parasitology

Veterinary Pathology

Veterinary Quarterly

Veterinary Radiology and Ultrasound

Veterinary Record

Veterinary Research (Pakistan)

Veterinary Research (BioMed Central)

Veterinary Research Communications

Veterinary Sciences Tomorrow

Veterinary Surgery

Veterinary Therapeutics

Veterinary World

West Indian Veterinary Journal

Wiener Tierärztliche Monatsschrift

Zoo Biology

Zoonoses and Public Health

## Competing interests

MC founded and co-ordinates the International Association of Veterinary Editors, which receives financial support from Wiley and Elsevier. MC is also the former Editor-in-Chief of *Veterinary Clinical Pathology*.

## Authors’ contributions

DG participated in the design of the study, drafted the questionnaire, analysed the data and drafted the manuscript. RD participated in the design of the study, helped to draft the questionnaire, coded data and helped to draft the manuscript. MB participated in the design of the study, helped to draft the questionnaire, coded data and helped to draft the manuscript. MC participated in the design of the study, tested the questionnaire, distributed the invitation to Editors-in-Chief and helped to draft the manuscript. All authors read and approved the final manuscript.

## Supplementary Material

Additional file 1 Questionnaire on reporting guidelines for veterinary Editors-in-Chief. Click here for file
