# Supplementary material for: A survey of the awareness, knowledge, policies and views of veterinary journal Editors-in-Chief on reporting guidelines for publication of research
Source: BMC Vet Res. 2014 Jan 10;10:10. doi: 10.1186/1746-6148-10-10 (PMC3922819; doi:10.1186/1746-6148-10-10)
Supplement: Additional file 1 — Questionnaire on reporting guidelines for veterinary Editors-in-Chief. [file 1746-6148-10-10-S1.docx]

**Additional file 1 – Questionnaire on reporting guidelines for veterinary Editors-in-Chief**

|  | Dear colleague,  This questionnaire is being carried out by the Centre for Evidence-based Veterinary Medicine at The University of Nottingham, UK, with the kind co-operation of Dr Mary Christopher of the International Association of Veterinary Editors (IAVE).  Our study aims to determine the extent of knowledge, and current views and policies, among Editors-in-Chief on reporting guidelines/standards for publication of research.  An invitation to fill out this questionnaire is being sent to all Editors-in-Chief on the IAVE circulation list. It is important that we get as complete a picture as possible across the full range of veterinary journals, so we would be most grateful if you could spare time from your busy schedule to fill out this short questionnaire.  **Please return a single reply as Editor-in-Chief on behalf of your journal, so we receive one response per journal. If there is more than one Editor-in-Chief, please submit a joint response.**  This research has received ethical approval from the School of Veterinary Medicine and Science Ethics Committee at The University of Nottingham. You may submit your response anonymously if you wish. However, it would help support that the survey is representative of the member journals in the International Association of Veterinary Editors if you provide the identifying data requested in Section 5. All information provided will be kept strictly confidential and no individual respondent will be identifiable in the published results. By completing this questionnaire you are indicating your consent to participate in the study.  The findings from this research may be published in peer-reviewed scientific journals and presented at conferences. Summary findings will be available to participants on request.  Thank you in advance for your participation.  Yours faithfully,  Douglas Grindlay BSc MA PhD MCLIP Marnie Brennan BSc (VB) BVMS PhD MRCVS Rachel Dean BVMS PhD DSAM (fel) MRCVS  Centre for Evidence-based Veterinary Medicine School of Veterinary Medicine and Science The University of Nottingham Sutton Bonington Campus Loughborough LE12 5RD UK  Phone: +44 (0)115 951 6743 Fax: +44 (0)115 951 6415 [www.nottingham.ac.uk/cevm](javascript:void(0))  **Please click on the ‘Start Survey’ button below to begin. The questionnaire should take between 10 and 20 minutes to complete. Questions marked with an asterix require an answer, due to the questionnaire logic. Please note that it is not possible to save and return to your response later.** |
| --- | --- |

| Section 1. |
| --- |
| This section is about your present knowledge of reporting guidelines/standards. |

| *1. Before receiving this questionnaire, did you know what a reporting guideline/standard was? (*Required) |
| --- |
| *Select one.* |
| \|  \| Yes \| (Go to question number 2.) \| \| --- \| --- \| --- \| \|  \| No \| (Go to question number 7.) \| |

| 2. How and where did you learn about reporting guidelines/standards? |
| --- |
| \|  \| \| --- \| \|  \| \|  \| \|  \| \|  \| |

| 3. Did you hear or read the presentation by Dr Eric von Elm on reporting guidelines/standards at the meeting of the International Association of Veterinary Editors in Geneva on 1 June 2010? |
| --- |
| *Select one.* |
| \|  \| Yes \| \| --- \| --- \| \|  \| No \| |

| 4. Are you aware of the Equator Network and its resources on reporting guidelines/standards? |
| --- |
| *Select one.* |
| \|  \| Yes \| \| --- \| --- \| \|  \| No \| |

| *5. Before receiving this questionnaire, were you aware of the existence of separate reporting guidelines/standards for different types of research study? (*Required) |
| --- |
| *Select one.* |
| \|  \| Yes \| (Go to question number 6.) \| \| --- \| --- \| --- \| \|  \| No \| (Go to question number 7.) \| |

| 6. Which of the following reporting guidelines/standards were you previously aware of? (Please tick any that apply, or choose "All of the above" or "None of the above") |
| --- |
| *Select all that apply.* |
| \|  \| ARRIVE (research using laboratory animals) \| \| --- \| --- \| \|  \| COGS (clinical guidelines) \| \|  \| CONSORT (randomised controlled trials) \| \|  \| COREQ (qualitative research) \| \|  \| GSPC/Gold Standard Publication Checklist (animal research studies) \| \|  \| MOOSE (meta-analyses of observational studies in epidemiology) \| \|  \| PRISMA (systematic reviews and meta-analyses) \| \|  \| REFLECT (randomised controlled trials for livestock and food safety) \| \|  \| STARD (diagnostic accuracy studies) \| \|  \| STREGA (genetic association studies) \| \|  \| STROBE (observational studies—cohort, case-control and cross-sectional) \| \|  \| TREND (non-randomised controlled trials) \| \|  \| None of the above \| \|  \| All of the above \| |

| Section 2. |
| --- |
| This section is about current and planned implementation of reporting guidelines/standards by your journal. If you are not sure about the answer to any of the questions, we would be grateful if you could obtain the necessary information from your editorial team.  NOTE FOR CLARITY: Reporting guidelines/standards are statements that provide advice on how to report research methods and findings for a particular type of study. They specify a minimum set of items required for a clear and transparent account of what was done and what was found. Examples are CONSORT for randomised controlled trials and PRISMA for systematic reviews and meta-analyses. |

| *7. Does your journal currently refer to any reporting guidelines/standards in its instructions to authors? (*Required) |
| --- |
| *Select one.* |
| \|  \| Yes \| (Go to question number 8.) \| \| --- \| --- \| --- \| \|  \| No \| (Go to question number 15.) \| \|  \| Don't know \| (Go to question number 16.) \| |

| 8. Which reporting guidelines/standards are referred to in the instructions to authors? |
| --- |
| \|  \| \| --- \| \|  \| \|  \| \|  \| \|  \| |

| 9. Why does your journal refer to these reporting guidelines/standards? |
| --- |
| \|  \| \| --- \| \|  \| \|  \| \|  \| \|  \| |

| 10. What does your journal do with submitted studies that do not follow the relevant reporting guideline/standard but otherwise satisfy its editorial criteria? |
| --- |
| \|  \| \| --- \| \|  \| \|  \| \|  \| \|  \| |

| *11. Does your journal have plans to implement additional reporting guidelines/standards in the future? (*Required) |
| --- |
| *Select one.* |
| \|  \| Yes \| (Go to question number 12.) \| \| --- \| --- \| --- \| \|  \| No \| (Go to question number 21.) \| \|  \| All relevant reporting guidelines/standards are already implemented \| (Go to question number 21.) \| \|  \| Don't know \| (Go to question number 21.) \| |

| 12. Which additional reporting guidelines/standards does your journal plan to implement? |
| --- |
| \|  \| \| --- \| \|  \| \|  \| \|  \| \|  \| |

| 13. What are the reasons for implementing these additional reporting guidelines/standards? |
| --- |
| \|  \| \| --- \| \|  \| \|  \| \|  \| \|  \| |

| *14. How does your journal plan to implement these additional reporting guidelines/standards? (*Required) |
| --- |
| *If answered, go to question number 21.* |
| \|  \| \| --- \| \|  \| \|  \| \|  \| \|  \| |

| *15. What are the reasons why your journal does not refer to reporting guidelines/standards in its instructions to authors? (*Required) |
| --- |
| \|  \| \| --- \| \|  \| \|  \| \|  \| \|  \| |

| *16. Does your journal have plans to implement reporting guidelines/standards in the future? (*Required) |
| --- |
| *Select one.* |
| \|  \| Yes \| (Go to question number 17.) \| \| --- \| --- \| --- \| \|  \| No \| (Go to question number 20.) \| \|  \| Don't know \| (Go to question number 21.) \| |

| 17. Which reporting guidelines/standards does your journal plan to implement? |
| --- |
| \|  \| \| --- \| \|  \| \|  \| \|  \| \|  \| |

| 18. How does your journal plan to implement these reporting guidelines/standards? |
| --- |
| \|  \| \| --- \| \|  \| \|  \| \|  \| \|  \| |

| *19. What are the reasons for implementing these reporting guidelines/standards? (*Required) |
| --- |
| *If answered, go to question number 21.* |
| \|  \| \| --- \| \|  \| \|  \| \|  \| \|  \| |

| *20. What are the reasons why your journal does not plan to implement reporting guidelines/standards in the future? (*Required) |
| --- |
| \|  \| \| --- \| \|  \| \|  \| \|  \| \|  \| |

| Section 3. |
| --- |
| This section is about your views on the potential need for reporting guidelines/standards and their implementation. |

| 21. Do you believe that reporting guidelines/standards should be adopted by all refereed veterinary journals where appropriate? |
| --- |
| *Select one.* |
| \|  \| Yes \| \| --- \| --- \| \|  \| No \| \|  \| Don't know \| |

| 22. What are the reasons for your answer to the previous question? |
| --- |
| \|  \| \| --- \| \|  \| \|  \| \|  \| \|  \| |

| 23. In your opinion, what factors may be preventing more widespread adoption of reporting guidelines/standards by veterinary journals? |
| --- |
| \|  \| \| --- \| \|  \| \|  \| \|  \| \|  \| |

| 24. In your opinion, what factors or actions would promote more widespread adoption of reporting guidelines/standards by veterinary journals? |
| --- |
| \|  \| \| --- \| \|  \| \|  \| \|  \| \|  \| |

| Section 4. |
| --- |
| This section is about information needs. |

| 25. Would you find it useful to have more information on reporting guidelines/standards? |
| --- |
| *Select one.* |
| \|  \| Yes \| \| --- \| --- \| \|  \| No \| |

| 26. What would be the best way(s) to disseminate such information to veterinary journal editors? |
| --- |
| \|  \| \| --- \| \|  \| \|  \| \|  \| \|  \| |

| 27. Do you have any further comments you would like to make about reporting guidelines/standards or about this survey? |
| --- |
| \|  \| \| --- \| \|  \| \|  \| \|  \| \|  \| |

| Section 5. |
| --- |
| This optional section is about your journal and about you. You do not need to answer any of these questions. However, information provided would help support that the responses received are representative of the member journals in the International Association of Veterinary Editors. If you provide your contact details, the information will be kept confidential. All results will be presented in an anonymised manner. |

| 28. OPTIONAL: What country is your journal officially published in? |
| --- |
| \|  \| \| --- \| |

| 29. OPTIONAL: The name of your journal |
| --- |
| \|  \| \| --- \| |

| 30. OPTIONAL: Your official role/job title in the journal |
| --- |
| \|  \| \| --- \| |

| 31. OPTIONAL: Your name |
| --- |
| \|  \| \| --- \| |

| 32. OPTIONAL: Your e-mail address—if you are interested in future studies |
| --- |
| \|  \| \| --- \| |

Thank you for your participation in this survey.

For further information about reporting guidelines and standards see the Equator Network website:
[www.equator-network.org](javascript:void(0))

If you wish to find out more about why we are doing this questionnaire, or have any queries about the questions, please contact Dr Douglas Grindlay at the address below.

Centre for Evidence-based Veterinary Medicine
School of Veterinary Medicine and Science
The University of Nottingham
Sutton Bonington Campus
Loughborough LE12 5RD
UK

Phone: +44 (0)115 951 6743
E-mail: douglas.grindlay@nottingham.ac.uk
[www.nottingham.ac.uk/cevm](javascript:void(0))
